# Effect of Online Reviews and Crowd Cues on Restaurant Choice of Customer: Moderating Role of Gender and Perceived Crowding

**DOI:** 10.3389/fpsyg.2021.780863

**Published:** 2021-12-02

**Authors:** Muhammad Asghar Ali, Ding Hooi Ting, Muhammad Ahmad-ur-Rahman, Shoukat Ali, Falik Shear, Muhammad Mazhar

**Affiliations:** ^1^Department of Management & Humanities, Universiti Teknologi PETRONAS, Seri Iskandar, Malaysia; ^2^Department of Management Sciences, National Textile University, Faisalabad, Pakistan; ^3^Department of Management Sciences, Riphah International University, Faisalabad, Pakistan

**Keywords:** online review, purchase intentions, review rating, situational cues, perceived crowdedness, gender, crowding

## Abstract

This study is aimed to identify the relative (direct) effect of online review ratings and perceived crowding on purchase intentions of a consumer. Our study also investigated the contingent effect of gender and perceived crowding between the relationship of exogenous and endogenous variables. This study was conducted in the Malaysian restaurant industry. We applied the purposive sampling technique to identify respondents, the mall intercept survey method was used for data collection. Smart PLS software was applied for data analysis (200 respondents). This study demonstrates through its results that online review ratings and perceived crowding have a positive effect on purchase intentions of a consumer. Moreover, if a consumer perceives crowding at a restaurant, this has a positive contingent effect on the relationship between review ratings and purchase intentions. This demonstrates that the consumer is more inclined to choose a restaurant with a high online review rating and has high perceived crowding at some unfamiliar place. Lastly, no evidence is found for the gender difference between review rating and purchase intentions; however, gender shows contingent effect and results confirmed that males preferred more crowded restaurants as compared to females. There are theoretical and practical implications for managers in the findings of this study.

## Introduction

Consumers are able to express their opinions and feelings about their experience of a product or service on online platforms, thanks to the significant growth of the internet and online networking sites. These online platforms (social media, websites, third-party review, etc.) play their role as a source of information for consumers in their decision-making about which product to use. The business scenario has been completely changed by this consumer reliance on the user-friendly mobile technology of internet for information search. Almost all restaurant businesses in Malaysia have adopted the internet as one of their primary communication methods to persuade the consumer through websites. To increase restaurant sales and win new patrons, companies have invested a significant portion of their budget in managing activities on online platforms.

Restaurants are most commonly selected by first-time customers who have done their research by visiting online platforms. They use the review ratings of different restaurants to make a risk-free choice that fits their taste and preferences (Park et al., 2007). In Malaysia, 83.2% of customers search for information about goods and services on the internet before making their purchase decisions (Department of Statistics Malaysia Official Portal, 2019). Because of this large majority, it is important to understand how online reviews influence decision-making of consumer (Gursoy et al., 2017). Electronic Word of Mouth (eWOM) refers to “any positive or negative statement” made by potential, actual, or former consumers about a product or company, which is made available to a multitude of people and institutions via the Internet (Roy et al., 2020). Existing customers create eWOM when they post product and service-related recommendations in the form of online reviews (Gottschalk and Mafael, 2017). These online reviews generate trust and purchase intentions, so they are considered an important source of information for consumers (Gharib et al., 2019; Grewal and Stephen, 2019; Punyatoya, 2019).

The sales of the product or service are enhanced or given an extra boost by positive online reviews with high star ratings because high online reviews influence perception of key facts of a shopper about product quality, value, and price (Chevalier and Mayzlin, 2006; Duan et al., 2008). Another experimental study in the context of hotel booking confirmed that the interaction effect of good review ratings and review relevance has a positive effect on booking intentions, while if the review rating is bad, the number of reviews does not have any effect on booking intentions (Gavilan et al., 2018).

Most of the studies highlighted the importance of online reviews by describing their role in the formation of consumer intentions to purchase (Gavilan et al., 2018; Gharib et al., 2019; Grewal and Stephen, 2019), but based on dual-processing theory by Vaisey (2009), consumers follow multiple information sources as part of the pre-purchase information search process. Consumers engage in a heuristic or systemic processing of information to evaluate available alternatives, depending on their level of involvement in the selection process. In restaurant selection, travellers generally only rely on heuristic cues and do not go for detailed/systematic information processing to decide on which restaurant to dine in. A consumer normally visits online review ratings (third-party sites, such as a trip advisor or other websites) to evaluate the reputation of the restaurant in contingent with offline cues as well, signalling the popularity of the service providers, and allowing the consumer to make a risk-free choice. This study focuses on online review ratings as a proxy of online reviews and perceived crowding as offline cues. The effect of crowding as one of the situational cues on consumer decision-making has been overlooked in previous literature (Mehta et al., 2013), but most of the time, people prefer to follow others with the belief that others have more authentic and useful information. Past studies show a diversity of outcomes for the effect of perceived crowding on consumer purchase behaviour (Huang et al., 2020; Santini et al., 2020; Thomas and Saenger, 2020). Some studies focussed on the negative aspects of crowding, such as causing anger, disgust, and anxiety (Machleit et al., 1994; Huang et al., 2018). Stress levels were also reported as affected by perceived crowding, as different levels of crowding cause different levels of stress, but evidence from past studies confirmed that crowding mitigates negative effects if the purpose of shopping is hedonic rather than utilitarian. Similarly, Baker and Wakefield (2012) posited crowding as the antecedent of positive emotions, excitement, and positive purchase behaviour. Crowding can be perceived by customers as a sign of high-quality food and the good reputation of the restaurant. The formation and changes in consumer attitudes and beliefs are explained by information integration theory by Anderson (1981), where consumers form their opinions based on prior knowledge about any product or service. The research population of this study had no prior experience with the respective restaurant, so they looked for different information sources to make a risk-free decision. At this early stage in the decision-making process, the source of information (for example, online reviews, and perceived crowding) provides clues regarding the reputation of a particular restaurant and influences purchase intentions negatively or positively. A consumer looking to choose a restaurant will go through online reviews and at the same time observe the number of diners at the particular restaurant. Similarly, information processing also varies with respect to gender (Arcand and Nantel, 2012). Studies have delineated that females give more attention to details while males use selective information cues to make an informed decision (Arcand and Nantel, 2012). Therefore, it will be interesting to identify how the effect of crowding and review rating will vary according to the gender difference. There is a scarcity of studies that investigated moderating role of gender difference in the relationship of perceived crowding and purchase intention, and online reviews and purchase intentions, respectively.

Hence, this study has three research objectives. The first objective is to identify the direct effect of online review ratings and perceived crowding on consumer purchase intentions. The second objective is to investigate the moderating effect of perceived crowding between the relationship of review ratings and purchase intentions. The third objective is to investigate the moderating role of gender between the relationship of review rating and purchase intentions, and crowding and purchase intentions, respectively.

This study makes four different contributions to the existing body of research. First, we investigated the effect of online review ratings and perceived crowding stimuli on purchase intentions, to analyse the influence of these online and offline information sources to better understand the consumer decision-making process. Secondly, previous studies have focussed on the direct effect of online reviews on purchase intentions, but this study also investigates the moderating role of perceived crowdedness in the relationship between review ratings and purchase intentions, which helps us to understand the consumer decision-making process from a different perspective. This study also investigates the contingent effect of gender, which helps us to understand how gender differences modify the effect of online reviews and perceived crowding on consumer decision-making (purchase intention). Fourthly, this study used a real-time survey, and data were collected from respondents (first-time visitors) while looking for a restaurant to dine in, in contrast to most of the past studies which used experimental methodology to investigate the direct effect of online reviews and perceived crowding on consumer purchases. We decided on this approach because experiments do not capture the real essence of a phenomenon due to their controlled environment and manipulation. Lastly, this study is specific to the Malaysian context. Malaysia is a collectivist society where values and consumer behaviour are different from Western society (which is more individualistic), so understanding consumer behaviour in a collectivist society was the focus of this particular study.

The latter part of this study consists of a literature review and hypothesis development. After the literature review section, the methodology section is presented along with the data collection procedure, and finally, the last part of this paper is dedicated to data analysis and contributions of the study.

## Literature Review

A consumer’s perception of crowding is considered vital in the retail service context for patronage decision and behavioural response. Most extant studies in the past identified the dark side of perceived crowdedness and confirmed its negative effects on consumption evaluation, intention to stay, and propensity to spend (Eroglu and Machleit, 1990; Blut and Iyer, 2020), but contrary to these findings, some other studies argued that perceived crowdedness has positive implications (Price et al., 1995; Huang et al., 2018). People gathering at a family function or on holidays can generate positive emotions due to the hedonic nature of these gatherings. Besides, varying levels of consumer perceived crowdedness are ascribed to differences in food quality, image, and price perception (Blut and Iyer, 2020). The highly crowded restaurant is perceived to have a good food quality, higher popularity among the public and seems to offer fair prices (Milman et al., 2020; Hou and Zhang, 2021). Similarly, at an unfamiliar place when the customer is uncertain about the quality of the restaurants, customers tend to choose the more crowded restaurant, believing that highly crowded restaurants offer better service and food quality.

### Review Rating

Thanks to advancements in technology, the consumer can obtain any type of information from online sources, such as sharing their experiences with other customers on the social networking site, such as Facebook, and third-party independent reviewer sites (Iqbal et al., 2021). The uncertain nature of the hospitality service sector drives consumers to look for tangible and intangible cues to get an idea about the service quality of a particular restaurant. A typical product review contains two types of information; the numerical rating is a quantitative summary of the experiences, attitudes, opinions, or sentiments of reviewer toward a product or service (Li et al., 2019), and the review text is an open-ended qualitative description of the opinions of the reviewer toward the product or service (Li et al., 2019). Previous studies demonstrate that consumers adjust their own choices according to the decisions made by other consumers (Wang and Yu, 2017). Similarly, for restaurant selection, online reviews or review ratings have been reported to play a vital role in restaurant selection before actual consumption (Kim and Tanford, 2019; Oliveira and Casais, 2019). Lack of prior experience with a restaurant pushes consumers to seek information from online reviews posted by friends, peers, and other consumers based on their experiences. Prevailing studies confirmed that a high review rating positively influences consumer attitudes, firm performance, (sales) and popularity perceptions (Chakraborty, 2019; Kakirala and Singh, 2020). The discussion shows that in an unfamiliar location, consumers evaluate review ratings as an indication of high-quality service before their actual purchase.

### Review Rating, Crowding, and Purchase Intentions

In general, when consumers have inadequate information, they follow decisions or views of other consumers to make inferences about the product or service quality of a restaurant so they can make a risk-free choice. Therefore, travellers search for online reviews on their mobile devices and choose the restaurant which best fits their needs, based on its review rating. When travellers are in an unfamiliar place and need to select a restaurant, they search for information about the restaurants in that area through mobile devices and decide based on review ratings. The higher the online review ratings, the more consumers are likely to choose that product or service (Chevalier and Mayzlin, 2006; Grewal and Stephen, 2019). However, online ratings are not the only source of information that a consumer uses to make his/her decisions, other information sources, such as crowding, can also influence the final choice of the consumer. Consumer perception of crowding is considered vital for patronage decisions and behavioural responses. Past literature has identified the dark side of perceived crowdedness and has confirmed that it negatively affects consumption evaluation, intention to stay, and propensity to spend (Huang et al., 2020; Santini et al., 2020; Thomas and Saenger, 2020). However, the effect of perceived crowding is not always negative – it usually depends on the type of event or activity in question and the personality traits of the customers (Mattila and Hanks, 2012). Crowdedness in a restaurant can be a sign of popularity, reasonable pricing, or good food quality, which is why crowdedness has been reported as a pivotal antecedent of purchase intentions (Thoo et al., 2018; Grewal and Stephen, 2019; Milman et al., 2020). Crowding is a common occurrence in the context of a restaurant, which consumers encounter at different stages of their decision-making process (Asghar Ali et al., 2021). Because of inconclusive research on crowding in the specific context of a restaurant, it will be interesting to investigate the moderating effect of perceived crowding on the relationship between online reviews and purchase intentions. Besides this, based on information integration theory, people change or update their attitudes and beliefs after integrating new information into their thought processes. In the marketing context, this theory suggests that online reviews engender positive attitudes and purchase intentions; however, a consumer’s observation of the crowdedness of a restaurant can also have a positive or negative effect on purchase intentions and final choice. Generally, people espouse social conformity (Asch, 1956), with each person believing that other people have comparatively more authentic information, resulting in herd behaviour. Herd theory in the context of choosing a restaurant would mean that when consumers are uncertain about restaurants in unfamiliar places, they choose a more crowded one, assuming that huge crowds in a restaurant indicate better quality of food, service, etc., therefore, relying on the others’ evaluation of the restaurant to arrive at a decision often in spite of the presence of information pointing to the contrary (Banerjee, 1992; Bikhchandani et al., 1992). We argue that with a higher or lower level of online review ratings, final choice of the restaurant of the consumer will be contingent on the level of the crowdedness of that restaurant. A consumer might choose a restaurant with a low online review rating and showing a high level of crowdedness, but the consumer may also choose a restaurant for which the review rating and crowdedness are both higher. Hence, at the last moment, crowdedness, instead of a more reliable source of information, can alter the final restaurant selection of the consumer.

Based on these assumptions, the following hypotheses are suggested:

H1: There will be a relationship between perceived crowding and consumer purchase intentions.

H2: There will be a relationship between review rating and consumer purchase intentions.

H3: Perceived crowding moderates the relationship between review rating and purchase intentions.

### The Moderating Role of Gender

Past studies have shown that males selectively process information to form judgements. Males follow heuristic information cues, which are openly available in the context. In contrast, females are more evaluative and make purposeful evaluations of the situation before making any judgement (Arcand and Nantel, 2012). Based on information processing, and difference in gender, a situation that seems very attractive for males maybe a security risk for females. Literature also supports perceived crowdedness having different implications for males and females who have different responses to the same situation (Machleit et al., 2000). For instance, in a crowded place, males may have more fun and fulfil their social needs while females might feel a risk, in the form of breach of personal space, depression, and helplessness, leading to a subsequent withdrawal from the situation (Regoeczi, 2008). Based on the above discussion, we propose that gender moderates the association between perceived crowding and purchase intentions, and that the effect is higher in males than females.

Moreover, the literature review confirms that positive online reviews positively influence purchase intentions (Ventre and Kolbe, 2020; Zhu et al., 2020). In particular, the importance of online reviews becomes more evident in an unfamiliar place, where the consumer visits a restaurant for the first time. Generally, in such situations, consumers rely on online reviews and access the information using mobile devices to make risk-free selections of restaurants that fit their tastes. The higher the online review ratings, the more likely consumers are to choose that restaurant (Chevalier and Mayzlin, 2006; Grewal and Stephen, 2019). The relationship between online reviews and purchase intentions has been reported to amplify or decline due to the influence of different moderators, such as product type, gender, need for cognition, weak tie eWOM, brand image, star category, and price (Chang and Chin, 2010; Lin et al., 2011; Wang et al., 2018; El-Said, 2020; Zhu et al., 2020).

However, there is a scarcity of studies dedicated to investigating the moderating role of gender for first-time visitors, in the context of a full-service restaurant. Past studies have shown mixed results regarding the contingent effect of gender on the association of online reviews and purchase intention. For instance, Kanungo and Jain (2012) confirmed that there is no gender difference between perceived risk and online purchase intentions; on the other hand, Sánchez-Torres et al. (2018) identified females rely more on online reviews than males before making a purchase decision. Similarly, another study confirmed females attach more importance to online information in contrast to males. Therefore, based on the above discussion we hypothesise:

H4: Gender moderates the relationship between perceived crowding and purchase intentions, and that this influence is stronger over males than females.

H5: Gender moderates the relationship between online review ratings and purchase intentions, and that this influence is stronger for males than females.

**FIGURE 1:**
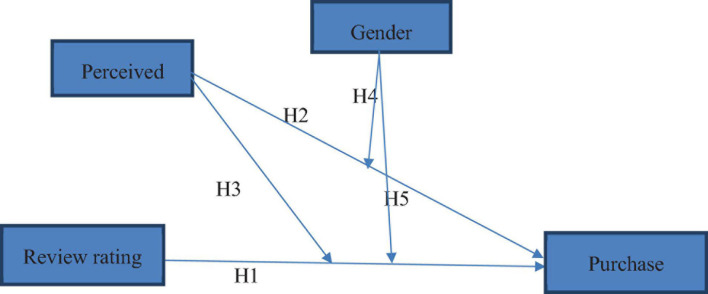
Conceptual framework.

We developed a conceptual framework (as shown in Figure 1) based on a detailed literature review, which depicts purchase intentions as an endogenous variable. Purchase intentions are the variable under study in this research work, while review rating and perceived crowding are the independent variables. Additionally, perceived crowding and gender have been proposed as moderators between the relationship of review rating and purchase intentions in this study.

## Methodology

### Population

The population used in this study consists of Malaysian people who dine in a restaurant at some unfamiliar place, and only the respondents who fulfilled the selection conditions set by the researcher were selected. The respondents must be visiting the selected restaurant for the first time and must have gone through online reviews before making their choice of restaurant. To see if the respondents met the selection criteria, they were first approached and told about the purpose of the study and then asked screening questions. “Is this your first-time visit to this place? Have you gone through online reviews?” All the respondents who answered these questions affirmatively were given a questionnaire for further data collection.

### Sampling Procedure and Data Collection

We used the purposive sampling technique for the identification and selection of respondents for this study. For data collection, the mall intercept technique was used, which is a survey method where respondents were intercepted in a social setting. This procedure includes interacting with the customer and asking screening questions before proceeding with the survey. For this study, the researcher first contacted the management of the restaurants, explained to them the purpose of this study, and asked for their permission for data collection. After getting their formal approval, the researcher contacted the customers who had just entered the respective restaurant, were sitting in its waiting area, and had not placed their order yet. The respondents were briefed about the purpose of the study and inclusion criteria before they were asked to fill in the questionnaire, starting with the preliminary questions. Qualified respondents who fulfilled the inclusion criteria were then requested to fill in the survey form regarding online review rating, perceived crowding, and purchase intention variables. The G power technique was applied to calculate the total sample size. For this study, the total sample size required was 129 at medium effect size (f^2^: 0.15 at 0.95 power). To make the results as general as possible, we distributed 250 questionnaires, but later, 50 questionnaires were discarded due to incomplete information. Therefore, the data of 200 respondents were used for further analysis using the Smart Partial Least Square Structural Equation Modeling (PLS-SEM) Technique.

Validated and reliable measurement instruments were adopted for perceived crowding, review rating, and purchase intentions. The measure of perceived crowding is adopted from Hui and Bateson (1991). The four-item scale measured on 7 points Likert scale was used. The measure of review rating and purchase intentions are adopted from Limayem et al. (2000) and Liu and Park (2015), respectively. The three items and five items scale, respectively, measured on 7 points Likert scale was used.

## Results

### Demographic Details

Demographic details demonstrate the majority of the respondents were Malay and Chinese. A detailed description of the demographics of the respondents is given in Table 1.

**TABLE 1:** Information of the participants.

Respondents’ profile	Percentage%
Gender	
Males	59
Females	41
Age	
Between 15 and 29	36.5
Between 30 and 39	31.5
Between 40 and 59	31.5
Between 50 and 60	0.5
Nationality	
Malaysian	100
Others	0
Race	
Malay	69.5
Chinese	30.0
Indian	0.5
Education	
Certificate	18.5
Diploma Degree	46.5
Masters	29.5
Ph.D.	5.5
Monthly Income	
RM 3000 to RM 3900	11.3
RM 4000 to RM 4900	28.2
RM 5000 to RM 5900	37.0
RM 6000 and above	23.5
Marital Status	
Single	65.5
Married	34.5

Smart PLS Software was used for data analysis, and the analysis section of this paper consists of two stages. In the first stage, to ensure the psychometric properties (validity and reliability) of the reflective measures, the researcher conducted confirmatory factor analysis (Ringle et al., 2015; Wang et al., 2021). In the second stage of data analysis, we employed structural analysis using the two-step approach recommended by Becker et al. (2012). To determine causality between independent and dependent variables and moderating effect, we used (SEM) Structural Model Analysis.

We employed Smart PLS-SEM due to its multiple advantages/benefits over covariance-based structural equation modeling (CB-SEM). Firstly, Smart PLS-SEM is preferred when a theory is at the initial level of development, and the aim is to predict the influence of independent variables on dependent variables. PLS-SEM is a second-generation multivariate analysis technique that is also useful for small sample sizes, and there is no specific condition regarding the normal distribution of data. In contrast to CB-SEM, PLS-SEM is ideally suitable for complex (reflective and formative) models, due to strong statistical power, and the ordinary least squares (OLS) method provides better results with elevated efficiency and higher R^2^ values, while decreasing the error term. These attributes and benefits make PLS-SEM a favoured method for data analysis when the aim of the researchers is to identify variance between endogenous and exogenous variables. Hence, we employed Smart PLS-SEM for this study (Hair et al., 2016, 2019).

Preliminary analysis was done before conducting a formal Smart PLS analysis and results depict there was no sign of any outlier, and data were normal within range of ±3 SD. Results of Harman’s one-factor test depict single factors account for 41.40% of variation and combining all components which are less than 50% (Podsakoff et al., 2003). This confirmed that there is no issue of common method bias (CMB) in the data.

### Reflective Measurement Model Analysis (Step 1)

This study also used a reflective measurement model. At the initial level, the reliability and validity of the measurement instrument were confirmed. For this purpose, the researcher takes into account important parameters, such as outer loading of the measurement items, CR, and average variance extracted. Past literature confirmed that the value of acceptable factor loading and composite reliability should be more than 0.7 for both, while values of Cronbach’s alpha and average variance extracted (AVE) should be more than 0.7 and 0.5 independently. Table 2 delineates that there is no issue of reliability and validity as results meet all minimum criteria (threshold values).

**TABLE 2:** Reflective measurement model.

Items	Outer loadings	VIF	p-Value	C. B. α	CR	AVE
Review Rating						
(RR1) The ratings helped me to learn about this restaurant.	0.802	1.36	0	0.779	0.871	0.69
(RR2) The ratings improved my understanding of the quality of this restaurant’s features.	0.834	1.95	0			
(RR3) The ratings were useful to evaluate the quality of this restaurant specifications/features.	0.859	1.95	0			

Perceived Crowding						
(Crd1) There was much traffic at the restaurant.	0.915	3.34	0	0.93	0.949	0.82
(Crd2) There were a lot of people at the restaurant	0.929	4.04	0			
(Crd3)The restaurant was a little too busy	0.876	3.54	0			
(Crd4) The restaurant seems very crowded to me	0.909	3.72	0			

Purchase Intentions						
(PI1) I prefer to purchase from this restaurant	0.823	3.29	0	0.894	0.913	677
(PI2) I am willing to purchase from this restaurant	0.887	4.04	0			
(PI3) I will make an effort to purchase from this restaurant	0.742	2.1	0			
(PI4) I would patronise this restaurant	0.855	2.81	0			
(PI5) I expect to purchase from this restaurant	0.8	2.29	0			

### Discriminant Validity

We tested discriminant validity using the Heterotrait-Monotrait (HTMT) approach and the Fornell-Larcker criterion. Henseler et al. (2015) suggested that the HTMT also known as the ratio of correlations. The HTMT value should always be lower than 0.90 to validate discriminant validity. Confirmed HTMT values of Table 3 are within an acceptable level, and this confirmed the discriminant validity. Furthermore, as per Fornell and Larcker (1981) to meet discriminant validity, “the square root of the AVE for each construct must be greater than its correlation with all other constructs,” hence Table 4 meets the same criteria and confirms discriminant validity.

**TABLE 3:** Fornell-Larcker criterion.

	Crowdedness	Purchase intentions	R. rating
Crowdedness	0.823		
Purchase Intentions	0.339	0.902	
Review rating	0.467	0.274	0.832

**TABLE 4:** Heterotrait-Monotrait ratio (HTMT).

	Crowdedness	Purchase intentions	R. rating
Crowdedness			
Purchase Intentions	0.404		
Review rating	0.546	0.306	

### Structural Model Measurement Analysis (Step 2)

Smart PLS was used for model estimation and moderation analysis, a method suggested by Hair et al. (2019). Table 5 shows the structural model results. We will evaluate the results using the standard coefficient, an effect size of 95%, a biased corrected confidence interval, and R^2^ values for further analysis. The results of the path analysis confirmed the proposed hypotheses H1 and H2 (main effect hypothesis). Review rating [H2: β: 0.389; (0.312, 0.504), *p*: 0.000] has a statistically significant positive main effect on purchase intentions. On the other hand, perceived crowding [H1: β: 0.283; (0.172, 0.414), *p*: 0.000] also explains the statistically significant variance in the dependent variable. Together, the variables of review rating and perceived crowding explain the 29.2% variance *R*^2^: 0.29.2 in the dependent variable. Effect size is another important indicator, depicting the magnitude of the effect of each independent variable on the dependent variable. The effect size of the review rating (*f*^2^: 0.198) is medium level and comparatively higher than perceived crowding (*f*^2^: 0.104). The last section reveals the moderating effect of perceived crowding and gender. The results in Table 5 demonstrate that perceived crowding positively moderates the relationship between review rating and purchase intentions, and our findings are a statistically significant hypothesis [H3: β: 0.130; (0.131, 0.272), *p*: 0.000] with effect size (*f*^2^: 0.058) and supported H3. Moreover, gender also moderates the relationship between perceived crowding and purchase intentions such an effect is high for males than females, these findings are statistically significant [H4: β: 0.215; (0.115, 0.307)], and supported H4. While, on the other hand, gender does not moderate the effect of review rating on purchase intentions, and results are statistically insignificant leads to the rejection of H5 [β: 0.128; (−0.040, 0.180)].

**TABLE 5:** Structural model assessment.

Hypothesis	STD coefficient (β)	SE	f^2^ Effect Size	t-values	P Value	95% confidence intervals	R^2^	Q^2^	Results
H1:Crowding→ Purchase intentions	0.283	0.067	0.104	4.567	0.00	[0.172,0.414]	0.292	0.154	Accepted
H2:Review Rating→ Purchase intentions	0.389	0.048	0.198	8.022	0.00	[0.312,0.504]			Accepted
H3:Crowding*Review rating	0.13	0.022		2.307	0.00	[0.113,0.272]	0.311		Accepted
H4:Crowding*Gender	0.215	0.155		2.160	0.03	[0.115,0.307]	0.644		Accepted
H5:Review rating*Gender	0.128	0.054		0.122	0.51	[−0.040,0.180]			Rejected

**FIGURE 2:**
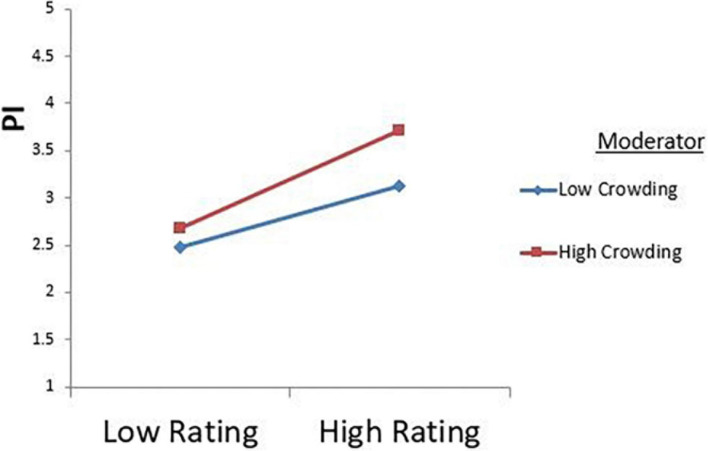
Moderation graph.

Figure 2 explains the role of the moderator in depicting the effect of the independent variable (the review rating) on the dependent variable (purchase intentions). For a restaurant with a low online rating, consumers are more likely to choose a highly crowded restaurant instead of a less crowded restaurant, as highlighted by the results. This shows that a restaurant with a low rating can still manage to attract customer intentions to purchase if they have enough customers present in the restaurant. On the other hand, a restaurant with a high review rating and a high level of perceived crowding will generate more positive intentions as compared to a restaurant with low crowding. Therefore, these results confirm that moving from a low rating to a high rating, and a gradual increase in the level of customer perceived crowding, would lead to higher purchase intentions, which would be to choose that restaurant to dine in.

**FIGURE 3:**
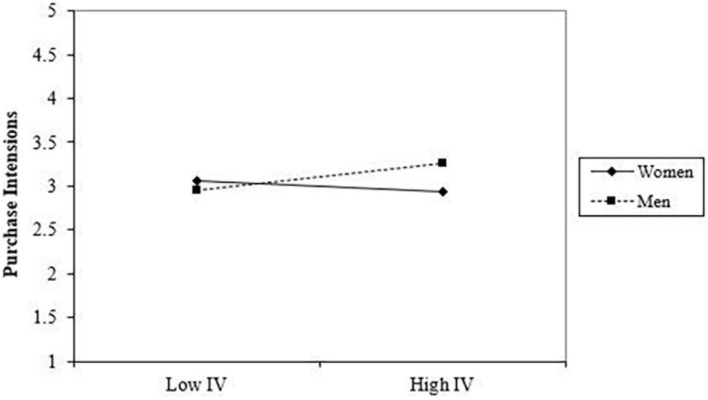
Moderation graph: IV = perceived crowding.

Moreover, Figure 3 explains the role of the moderator in depicting the effect of the independent variable (perceived crowding) on the dependent variable (purchase intentions). For a restaurant with less crowding, females are more likely to have higher purchase intention as compared to males, as highlighted in the results. This shows that a restaurant with less crowding can still manage to attract the females’ intentions to purchase because females feel comfortable in a less crowded place in the restaurant. On the other hand, a restaurant with a high level of crowdedness will create more positive purchase intentions in males as compared to females. Therefore, these results confirm, with a gradual increase in the level of customer perceived crowding, males would lead to higher purchase intentions to choose that restaurant to dine in, while females would be reluctant to choose that restaurant with low purchase intention.

## Discussion, Conclusion, and Implication

The results of this study show that online review ratings, perceived crowding, and gender are important factors in consumer restaurant choice (purchase intentions) in an unfamiliar place. Moreover, perceived crowding also has a moderating effect and positively increases the influence of online review ratings on the purchase intentions of a consumer. Gender also moderates between the relationship of perceived crowding and purchase intentions such that the relation is stronger for males than females.

Information integration theory and dual processing theory both explain the phenomenon of consumer restaurant choice in an unfamiliar place. While selecting a restaurant to dine in, consumers choose different methods to find information about a product or service, such as online reviews (star ratings) and the behaviour of other consumers (crowding), which prove to be vital sources of information for risk-free decision-making (Thoo et al., 2018; Yi et al., 2018; Milman et al., 2020; Hou and Zhang, 2021). Although perceived crowding has a positive association with purchase intention, however, this effect varies with respect to gender (higher for males and lower for females).

A online review rating of consumer has a significant influence on consumer choice, as confirmed by the results. A high online rating is considered a sign of decent service and food quality, resulting in a positive attitude and motivating people to prefer the high-rated restaurant. These findings are in line with a previous study on consumer decision-making by Sparks and Browning (2011), they conducted an experimental study and confirmed that consumers tend to rely on easily processed information, showing that positive reviews and numerical review rating positively influence hotel booking intentions and trust. Similarly, we can generalise the results of this study to the whole restaurant industry of Malaysia. The findings of this study also confirm that although online reviews are important, the consumers do not only depend on review ratings when choosing a restaurant, since offline cues, such as crowding, can also affect their final choice. Crowdedness in retailing has been reported to create stress, intentions to spend less time, and money and avoidance behaviour. However, in the context of restaurants, the results of crowding are contrary to past studies and positively affect purchase intentions. In this scenario, consumers prefer to join a crowded restaurant, believing it is a signal of popularity and better service quality. These results are in line with previous research conducted by Ha et al. (2016).

The interaction effect of perceived crowding and online review rating significantly predicts consumer purchase intentions. Different levels of crowding, for example, low or medium crowding, have shown interesting effects on consumer choices, since consumers were particularly more inclined to select a restaurant with high crowding as compared to a low-crowded restaurant. Moreover, intention of a consumer to choose a particular restaurant is high for a highly rated and high-crowded restaurant as compared to a highly rated but low-crowded restaurant. On the other hand, for low-rated restaurants, purchase intentions of consumer were significantly higher for a high-crowded restaurant instead of a low-crowded restaurant. These findings are consistent with Ha et al. (2016) they conducted a scenario-based experimental study and confirmed that consumers follow each other’s choices and opinions, and also identified that crowdedness positively influences intentions of consumer to dine in a restaurant in an unfamiliar place. The study further suggests that consumers follow both review rating and crowdedness for dine-in and take away choices. Similarly, in the Malaysian context, the interactive effect of crowding and review rating confirmed that consumers follow both cues for restaurant selection. This implies that a high review rating and the presence of other patrons are believed to be an indication of restaurant popularity, credibility and fair prices, which compels the customer to choose the crowded restaurant. In addition, the effect of crowdedness on restaurant choice is higher for males in contrast to females. This is a novel contribution of this study to the existing body of literature because up to the knowledge of the researchers no study investigated moderating role of gender between the relationship of perceived crowding and purchase intentions Lastly, the moderating role of gender on the relationship between review ratings and customer purchase intentions to dine is insignificant. Contrary to these findings, past studies have confirmed significant gender differences between the association of online reviews and online purchase intentions, in which females trust online reviews more than males and have high purchase intention. This implies that in the context of restaurant choice, gender does not cause any difference in the relationship between online reviews and purchase intentions. The findings of this study have both theoretical and practical implications.

### Theoretical Implications

This study is based on information processing theory and herd theory and has demonstrated important theoretical contributions. The first findings demonstrate that high online review ratings develop high purchase intentions, and these findings are in line with previous studies on consumer decision-making (Dellarocas, 2003; Zhang et al., 2010). However, more importantly, due to the interactive effect of crowding and review rating, this study found that if the review rating is low, crowdedness takes the lead in influencing consumer purchase intentions. Specifically, the consumer will more probably choose a restaurant with high crowding, which suggests that even though both stimuli are critical, a last-minute observation on the crowdedness of the restaurant can have a stronger influence on their intention to choose the restaurant than the online ratings, especially when the review rating is low. This depicts herd behaviour, where consumers follow behaviour of other consumers for the choice of restaurant. Under uncertain conditions, crowdedness works as an indication of restaurant popularity.

Secondly, this study provides the unique implication that the interaction effect between restaurant ratings and crowdedness is at a high level. The intentions of consumers to patronise the restaurant would be highest for a restaurant that is both high on review rating and has a high level of crowdedness. Hence, the transition of a restaurant from a low level of crowding to a high level of crowding positively influences consumer purchase intentions. Therefore, this is an important theoretical contribution to the existing body of knowledge on consumer decision-making.

Finally, gender as a moderator has been investigated in many studies in different contexts (Kolyesnikova et al., 2009; Heidarian, 2019; Huang and van der Veen, 2019). However, the identification of gender as a significant moderator in the effect of crowding on purchase intentions, and finding out that this is higher for males as compared to females, provides a unique theoretical contribution as this link was never investigated in the Malaysian restaurant context.

### Managerial Implications

This study has important practical implications for the managers of restaurants. Firstly, the study demonstrates that online reviews/ratings are a vital source of information, and the results of the study depict their significant direct effect on consumer purchase intentions. A higher online rating is a desirable goal of every company or restaurant, but negative reviews adversely affect or influence reputation of a company and may make it fail to attract new customers. Review/online ratings of a consumer are based on their experience with the company, but negative reviews are easy to make due to unaccountability and anonymity.

To establish a high star rating, zero-defect service should be the policy of the company, and in case of a failure of service, problems should be resolved with proper compensation on the spot. This policy shift will decrease the probability of customers posting negative review ratings. Moreover, delighted customers should be encouraged to give their opinion or rating online, which will increase the online rating of the restaurant and help to win the trust of new customers.

Moreover, restaurant managers should hire experts to manage the online information flow of review ratings, to help the company identify and eliminate fake reviews posted by unknown people or by competitors. Moreover, experts can also calm annoyed customers by giving positive replies and showing concern for the issues posted in negative online reviews and ratings. Reply with a proper apology will reduce the negative emotions of the consumers and will generate re-patronage intentions with the result that consumers will delete or change negative remarks to positive comments, which will be useful for attracting new customers.

Secondly, the managers of the restaurants should manage both online review ratings and the level of crowdedness at the same time, since the results of the study demonstrate that due to the easy access of the internet, the majority of consumers in unfamiliar places look for online reviews/ratings, and their intention to dine in a restaurant also depends on the crowdedness of the restaurant. Consumers prefer highly crowded restaurants in both high and low rating situations. Although star rating is an important indicator of consumer purchase intentions, since consumers always have multiple options available online, in an unfamiliar place the consumer himself might be looking for a crowded restaurant to dine in. Hence, despite the high online rating, offline conformity of information in the form of crowdedness is necessary to make a risk-free decision. Moreover, to manage restaurant crowdedness, employees should be provided a suitable working environment with proper training, as a toxic work environment negatively influences work engagement of employees (Rasool et al., 2021; Zhou et al., 2021). In addition to ensure sustainable performance organisation should adopt proper human resource management practices to achieve its long-term objectives (Rasool et al., 2019).

It is also important for restaurant managers to make sure that a certain number of consumers are present at the restaurant during peak hours. People follow the behaviour of the crowd, although the presence of other consumers is considered as a sign of popularity and good food quality, it has different implications for males and females. The presence of other customers will attract the attention of prospective male customers looking for a restaurant to dine in, and there is a high probability that in such a situation the consumer will choose a more crowded restaurant compared with a less crowded one. The situation is different for female customers, as females have shown a tendency to not choose a crowded restaurant. Females are an important segment of the Malaysian population distribution where the female percentage (51%) is higher than males (49%) (Department of Statistics Malaysia Official Portal, 2019).

Restaurant managers should take into account this important demographic element and develop restaurant strategies accordingly. For instance, restaurant managers can make design and seating arrangements, and allocate appropriate space in such a way that it suits the requirements of male and female customers concurrently. For instance, patrons (both males and females) could be seated along with the windows or in other visible parts of the restaurant to attract new customers, and there should be adequate space between the tables with proper walkaways so that female customers might not feel a breach of personal space and feel comfortable and confident during their visit. Moreover, special meal discounts, offers or, loyalty cards should be announced and offered to existing customers. This strategy will be beneficial in maintaining existing customers in the restaurant and will be useful in attracting new customers.

### Limitations

This study does have some limitations. Firstly, although this is a real-time study, the data are collected at a single point in time (the cross-sectional study), and the same framework can be used in a longitudinal study to identify fluctuations in consumer behaviour for the restaurant choice process. Secondly, we used the purposive sampling technique to identify respondents, so future studies can use a probability sampling technique to make the results more generalised. Thirdly, the literature suggests that online reviews do not only consist of review ratings but also have multiple characteristics, such as review volume, review relevance, and review credibility. Future studies can be used to check the comparative effect of other online review characteristics on consumer purchase behaviour. Moreover, the role of crowding can also be explored in other areas, such as gambling, tourism, and sports.

## Data Availability Statement

The original contributions presented in the study are included in the article/supplementary material, further inquiries can be directed to the corresponding author.

## Author Contributions

MAA: conception or design of the work and drafting the article. MAA and DT: data collection. MA-U-R: data analysis and interpretation. SA: facilitate in critical revision of the article. FS and MM: helped in proofreading to improve the quality of the manuscript. All authors contributed to the article and approved the submitted version.

## Conflict of Interest

The authors declare that the research was conducted in the absence of any commercial or financial relationships that could be construed as a potential conflict of interest.

## Publisher’s Note

All claims expressed in this article are solely those of the authors and do not necessarily represent those of their affiliated organizations, or those of the publisher, the editors and the reviewers. Any product that may be evaluated in this article, or claim that may be made by its manufacturer, is not guaranteed or endorsed by the publisher.
